# Continuous *versus* bolus norepinephrine administration to treat hypotension after induction of general anaesthesia in low-to-moderate risk noncardiac surgery patients: a randomised trial

**DOI:** 10.1016/j.bja.2025.03.017

**Published:** 2025-02-05

**Authors:** Kristen K. Thomsen, Finn Külls, Christina Vokuhl, Linda Krause, Dominik Müller, Max Bossemeyer, Mirja Wegge, Alina Kröker, Alina Bergholz, Christian Zöllner, Daniel I. Sessler, Bernd Saugel

**Affiliations:** 1 Department of Anesthesiology, Center of Anesthesiology and Intensive Care Medicine, University Medical Center Hamburg-Eppendorf, Hamburg, Germany; 2 Outcomes Research Consortium®, Houston, TX, USA; 3 Institute of Medical Biometry and Epidemiology, University Medical Center Hamburg-Eppendorf, Hamburg, Germany; 4 Center for Outcomes Research and Department of Anesthesiology, UTHealth, Houston, TX, USA

**Keywords:** anaesthesia induction, arterial pressure, cardiovascular dynamics, general anaesthesia, haemodynamic monitoring, norepinephrine, vasopressor

## Abstract

**Background:**

Hypotension after induction of general anaesthesia (postinduction hypotension) is common in patients undergoing noncardiac surgery and frequently requires treatment with vasopressors such as norepinephrine. We tested the hypothesis that giving norepinephrine continuously using a syringe infusion pump, compared with giving it as repeated manual boluses, reduces postinduction hypotension within 15 min after starting induction of general anaesthesia in low-to-moderate risk noncardiac surgery patients.

**Methods:**

Patients undergoing elective noncardiac surgery were randomised to either continuous norepinephrine infusion or manual bolus norepinephrine administration intravenously during induction of general anaesthesia. In both groups, norepinephrine was administered through a peripheral venous catheter. Blood pressure was measured by clinicians using intermittent oscillometry. We additionally performed blinded continuous noninvasive blood pressure monitoring to quantify the duration and extent of postinduction hypotension. The primary endpoint was postinduction hypotension, defined as the area under a MAP of 65 mm Hg within 15 min after starting induction of general anaesthesia.

**Results:**

From 276 randomised patients, 261 had complete data (median age: 62 yr; 40% female). The median (25th–75th percentile) area under a MAP of 65 mm Hg was 3.6 (0.0–16.6) mm Hg × min in patients assigned to continuous norepinephrine infusion, compared with 5.5 (0.5–24.5) mm Hg × min in patients assigned to manual bolus norepinephrine administration (*P*=0.070). The median duration of MAP values <65 mm Hg was 1.0 (0.0–2.5) min *vs* 1.4 (0.2–3.2) min (*P*=0.052).

**Conclusions:**

Continuous administration of norepinephrine, compared with repeated manual bolus doses, did not reduce postinduction hypotension in low-to-moderate risk noncardiac surgery patients who had intermittent oscillometric blood pressure monitoring.

**Editor's key points**

• Hypotension after induction of general anaesthesia is common in noncardiac surgery and might be associated with harm.
• This RCT tested the hypothesis that giving norepinephrine via infusion *vs* repeated manual boluses reduces hypotension within 15 min of inducing general anaesthesia.
• A total of 276 low-to-moderate risk patients undergoing elective noncardiac surgery were randomised.
• Continuous norepinephrine infusion failed to reduce hypotension after induction of general anaesthesia compared with manual boluses.
• Hypotension after induction of general anaesthesia is not affected by the mode of delivering norepinephrine in low-to-moderate risk patients undergoing elective noncardiac surgery.

Hypotension is common in patients undergoing noncardiac surgery with general anaesthesia and is associated with acute kidney injury,^1^, ^2^, ^3^, ^4^, ^5^ myocardial injury,^2^ and myocardial infarction.^3^^,^^6^^,^^7^ Organ injury is a function of hypotension severity and duration. On a population basis, the hypotensive harm threshold for organ injury appears to be a MAP <60–65 mm Hg.^2^ Consensus recommendations are thus to keep MAP >60–65 mm Hg in patients undergoing noncardiac surgery.^8^^,^^9^

Notably, about one-third of hypotension occurs between induction of general anaesthesia and surgical incision.^10^ It is reasonable to assume that postinduction hypotension can be limited by careful anaesthetic management, including administration of vasopressors such as phenylephrine or norepinephrine to treat anaesthetic-induced vasodilation.^11^ During anaesthetic induction, norepinephrine is usually given as repeated manual boluses of 5, 10, or 20 μg. Norepinephrine boluses rapidly increase blood pressure, but its half-life is short.^12^ Consequently, well-timed repeated boluses are often necessary.

Blood pressure is usually measured only intermittently during anaesthetic induction in low-to-moderate risk noncardiac surgery patients. Consequently, clinicians may belatedly recognise hypotension when the effect of a norepinephrine bolus begins to diminish. Our theory therefore was that continuous, instead of bolus, administration of norepinephrine during anaesthetic induction may help reduce postinduction hypotension in low-to-moderate risk noncardiac surgery patients. Specifically, we tested the hypothesis that giving norepinephrine continuously using a syringe infusion pump, compared with giving it as repeated manual boluses, reduces postinduction hypotension within 15 min after starting induction of general anaesthesia in male and female low-to-moderate risk noncardiac surgery patients who have intermittent oscillometric blood pressure monitoring.

## Methods

### Study design

The ‘ContInuous versus bolus Norepinephrine administration to treat postinDUCTion hypotension in low-to-moderate risk non-cardiac surgery patients - (INDUCT) trial’ was performed at the Department of Anesthesiology, Center of Anesthesiology and Intensive Care Medicine, University Medical Center Hamburg-Eppendorf between July 2023 and June 2024. The trial was approved by the ethics committee (Ethikkommission der Ärztekammer Hamburg, Hamburg, Germany, registration number 2023-100999-BO-ff) on February 27, 2023, and registered at ClinicalTrials.gov (NCT05940649) on July 4, 2023. Participating patients provided written informed consent. We report the trial according to the Consolidated Standards of Reporting Trials (CONSORT) statement.^13^

### Inclusion criteria

We included consenting patients scheduled for elective noncardiac surgery with general anaesthesia who were at least 45 yr old and designated ASA physical status 2 or higher. Enrolment was restricted to patients in whom intermittent oscillometric blood pressure monitoring was planned during anaesthetic induction and surgery.

### Exclusion criteria

We excluded patients undergoing emergency or transplant surgery, patients who previously underwent organ transplant surgery, and patients in whom finger-cuff blood pressure monitoring was not possible. We also excluded patients who were pregnant, were not in sinus rhythm, or in whom rapid sequence induction was planned.

### Clinical care

Patients were equipped with standard anaesthetic monitoring, including electrocardiography, pulse oximetry, and upper-arm cuff oscillometry (Infinity Delta patient monitors; Dräger Medical, Lübeck, Germany; or IntelliVue MX750; Philips, Amsterdam, The Netherlands). For study purposes, we also continuously monitored blood pressure using a finger-cuff system (CNAP system; CNSystems Medizintechnik, Graz, Austria) (details below). Anaesthesiologists were blinded to continuous finger-cuff blood pressure monitoring and thus managed blood pressure based on intermittent oscillometric measurements at 2.5-min intervals per clinical routine. A peripheral venous catheter for norepinephrine administration was inserted on the arm opposite to the arm used for upper-arm cuff oscillometry.

In both groups, general anaesthesia was induced using an opioid (sufentanil, remifentanil, or fentanyl) and propofol. In patients requiring tracheal intubation, a neuromuscular blocking agent (rocuronium, mivacurium, or succinylcholine) was administered. Patients' lungs were mechanically ventilated via a tracheal tube or laryngeal mask. General anaesthesia was maintained with a continuous propofol infusion or inhaled sevoflurane. Anaesthesiologists were asked to maintain MAP >65 mm Hg per institutional routine. Balanced crystalloids were administered at the discretion of the attending anaesthesiologist.

### Randomisation and blinding

Patients were randomised to continuous norepinephrine infusion via a syringe infusion pump or to manual bolus norepinephrine administration in a 1:1 ratio without blocking or stratification based on computer-generated codes. Group allocation was concealed in sequentially numbered opaque envelopes that were opened shortly before anaesthetic induction. Patients were blinded to group allocation, but anaesthesiologists could not be.

### Study interventions

In patients assigned to continuous norepinephrine infusion, norepinephrine was administered via a syringe infusion pump (Perfusor Space; B Braun, Melsungen, Germany) using a 50-ml syringe containing 3 mg norepinephrine diluted with normal saline 0.9% to 50 ml (resulting in a concentration of 60 μg ml^−1^). In clinical practice, infusion rates during anaesthetic induction typically range between 0.03 μg kg^−1^ min^−1^ and 0.2 μg kg^−1^ min^−1^. The norepinephrine syringe was connected to an i.v. infusion line via a three-way stopcock before induction of anaesthesia. Anaesthesiologists were free to start and adjust the norepinephrine infusion rate as needed and to give additional norepinephrine boluses via the syringe infusion pump.

In patients assigned to manual bolus norepinephrine administration, norepinephrine was manually given as boluses from a 10-ml syringe containing 100 μg norepinephrine (resulting in a concentration of 10 μg ml^−1^) at the discretion of the treating anaesthesiologist.

### Blinded continuous blood pressure monitoring

To quantify postinduction hypotension, we performed blinded continuous blood pressure monitoring using the finger-cuff CNAP system, which has been validated against intraarterial blood pressure monitoring in patients undergoing noncardiac surgery.^14^ An appropriate finger-cuff (small, medium, or large) was positioned on the proximal phalanx of the third or fourth finger of the arm contralateral to that used for upper-arm cuff oscillometry. We manually recalibrated the CNAP to every new oscillometric value.

Continuous finger-cuff blood pressure measurements were extracted from the CNAP system in 1-s intervals. We excluded artifactual pressures using the following sequential rules: (1) blood pressures identified as artifacts by trial personnel; (2) systolic arterial pressures >280 mm Hg or <30 mm Hg; (3) systolic arterial pressures below diastolic arterial pressures plus 5 mm Hg; or (4) diastolic arterial pressures >150 mm Hg or <10 mm Hg. Excluded blood pressure values were replaced by the closest adjacent values.

### Primary endpoint

The primary endpoint was postinduction hypotension quantified as the area under a MAP of 65 mm Hg (mm Hg × min) within 15 min after starting induction of general anaesthesia (Supplementary Fig. S1). The area under a MAP of 65 mm Hg was calculated by subtracting MAP measurements from 65 mm Hg, multiplying positive differences with the time difference (in minutes) between this MAP measurement and the consecutive MAP measurement, and summing the values.

### Secondary endpoints

We also assessed the areas under MAP values of 60, 50, and 40 mm Hg and above MAP values of 100, 110, 120, and 140 mm Hg; the absolute number and fraction of patients who had at least one MAP less than 65, 60, 50, and 40 mm Hg and higher than 100, 110, 120, and 140 mm Hg; the durations of MAP values less than 65, 60, 50, and 40 mm Hg or higher than 100, 110, 120, and 140 mm Hg; and absolute numbers and fractions of patients who had MAP values less than 65, 60, 50, and 40 mm Hg for at least 1 continuous minute. We also assessed the cumulative dose of norepinephrine patients were given, indexed to actual body weight. On a reviewer's request, we analysed *post hoc* the norepinephrine infusion rate, the median number of norepinephrine infusion rate adjustments made, the median number of norepinephrine boluses given, and the median norepinephrine bolus dose.

### Statistical analysis

The statistical analysis plan was part of the study protocol and filed with the ethics committee before data were accessed. Patients' demographic, baseline, and clinical characteristics are described separately for patients assigned to continuous norepinephrine infusion and to manual bolus norepinephrine administration. Categorical data are presented as absolute numbers and percentages. Continuous data are presented as medians (25th–75th percentile).

The primary endpoint was analysed using a two-sample Wilcoxon rank-sum test with a corresponding 95% confidence interval (CI), and an estimator for the difference of the location parameters was computed. Of note, the estimator for the difference in location parameters is not the difference in medians, but instead the median of differences between a sample from one group and a sample from the comparison group. A significance level of 5% was applied for the primary endpoint. Continuous secondary endpoints were analysed analogously to the primary endpoint. Binary secondary endpoints were analysed using Fisher's exact test for count data.

### Sample size

Based on pilot data,^15^ we expected that the mean (standard deviation [sd]) of the area under a MAP of 65 mm Hg would be 40 (sd 50) mm Hg × min in patients randomised to manual bolus norepinephrine administration. Therefore, a total of 262 patients (*n*=131 patients per group) would provide 90% power at a significance level alpha of 0.05 to detect a reduction in the area under a MAP of 65 mm Hg of 20 mm Hg × min or more by continuous norepinephrine infusion. We expected a dropout rate of 5% and therefore planned to enrol a total of 276 patients, with 138 patients each assigned to continuous norepinephrine infusion or to manual bolus norepinephrine administration.

## Results

### Study participants

**Fig 1:**
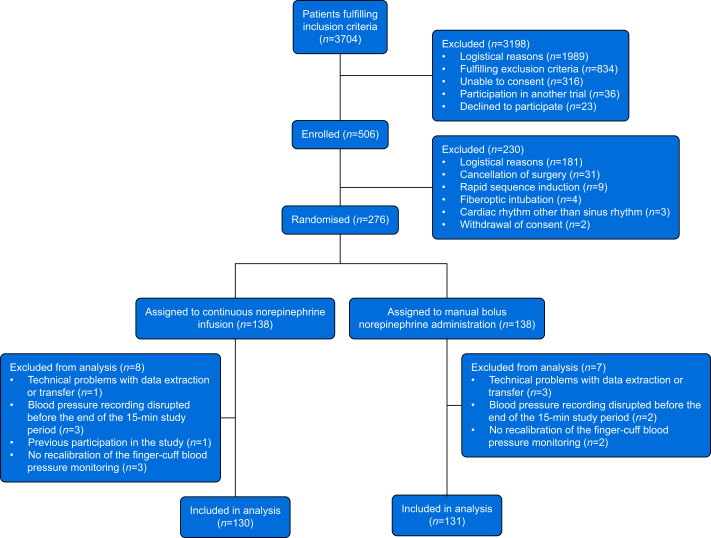
Flow chart illustrating patient screening, enrolment, randomisation, and reasons for exclusion.

We randomised 276 patients but excluded 15 patients due to incomplete blood pressure recordings, calibration failure and/or inability to extract the raw data (Fig. 1). We thus included 261 patients with complete data recordings in the final analysis, 130 assigned to continuous norepinephrine infusion and 131 assigned to manual bolus norepinephrine administration (Table 1). Anaesthetic management was similar in both groups (Supplementary Table S1).

**Table 1:** Demographic and baseline characteristics. Data are presented as median (25th–75th percentile), or absolute number (percentage), *n* (%).

		Continuous norepinephrine infusion (*n*=130)	Manual bolus norepinephrine administration (*n*=131)
Age (yr)		62 (55–72)	63 (56–70)
Height (cm)		173 (168–179)	176 (170–180)
Weight (kg)		79 (70–92)	80 (72–91)
Body mass index (kg m^−2^)		25 (24–29)	26 (23–29)
Sex	Female	59 (45)	45 (34)
	Male	71 (55)	86 (66)
ASA physical status	2	99 (76)	94 (72)
	3	31 (24)	37 (28)
Chronic arterial hypertension		54 (42)	73 (56)
Chronic obstructive pulmonary disease		6 (5)	8 (6)
Diabetes mellitus		10 (8)	16 (12)
Chronic heart failure		0 (0)	0 (0)
Liver disease		5 (4)	1 (1)
Chronic kidney disease		2 (2)	6 (5)
Coronary artery disease		7 (5)	9 (7)
Cerebrovascular disease		3 (2)	0 (0)
Otorhinolaryngology		32 (25)	34 (26)
Oral and maxillofacial surgery		21 (16)	21 (16)
Gynaecological surgery		21 (16)	10 (8)
Urological surgery		53 (41)	64 (49)
Orthopaedic surgery		0 (0)	1 (1)
Plastic surgery		3 (2)	1 (1)

### Primary endpoint

**Fig 2:**
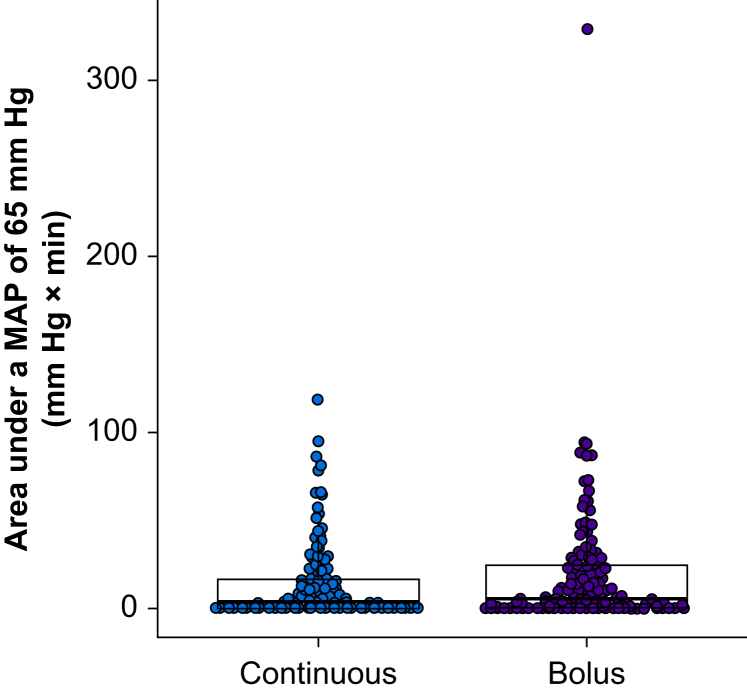
Boxplots with overlaying scatter plots illustrating areas under a mean arterial pressure (MAP) of 65 mm Hg in patients assigned to continuous norepinephrine infusion and manual bolus norepinephrine administration during induction of anaesthesia.

Within 15 min of induction of general anaesthesia, the median area under a MAP of 65 mm Hg was 3.6 (0.0–16.6) mm Hg × min in patients assigned to continuous norepinephrine infusion, compared with 5.5 (0.5–24.5) mm Hg × min in patients randomised to manual bolus norepinephrine administration (*P*=0.070; estimated location shift –0.51 [95% CI –2.71 to 0.000087] mm Hg × min; Fig. 2).

### Secondary endpoints

**Fig 3:**
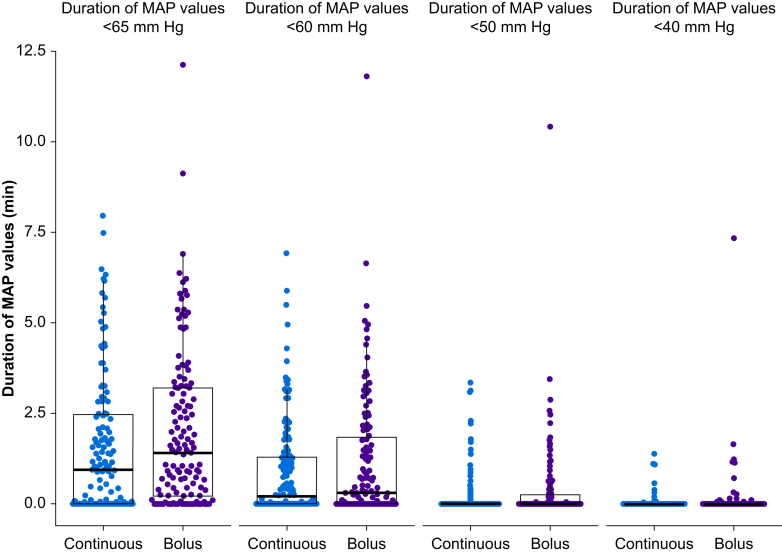
Boxplots with overlaying scatter plots illustrating the duration of mean arterial pressure (MAP) values less than 65, 60, 50, and 40 mm Hg in patients assigned to continuous norepinephrine infusion and manual bolus norepinephrine administration during induction of anaesthesia.

Ninety (69%) patients assigned to continuous norepinephrine infusion and 107 (82%) patients assigned to manual bolus norepinephrine administration had at least one MAP less than 65 mm Hg (*P*=0.022; Table 2). The median duration of MAP values <65 mm Hg was 1.0 (0.0–2.5) min in patients assigned to continuous norepinephrine infusion and 1.4 (0.2–3.2) min in patients assigned to manual bolus norepinephrine administration (*P*=0.052; estimated location shift –0.21 [95% CI –0.64 to 0.000072] min; Fig. 3).

**Table 2:** Secondary endpoints quantifying hypotension. Categorical data are presented as absolute number (percentage) and continuous data as median (25th–75th percentile). MAP, mean arterial pressure. ∗*P*-values correspond to Wilcoxon rank-sum tests with continuity correction. ^†^*P*-values correspond to Fisher's exact test for count data.

Outcome	Continuous norepinephrine infusion (*n*=130)	Manual bolus norepinephrine administration (*n*=131)	*P*-values
Area under a MAP of 60 mm Hg, mm Hg × min	0.4 (0.0–6.5)	0.9 (0.0–12.7)	0.222∗
Area under a MAP of 50 mm Hg, mm Hg × min	0.0 (0.0–0.0)	0.0 (0.0–0.6)	0.075∗
Area under a MAP of 40 mm Hg, mm Hg × min	0.0 (0.0–0.0)	0.0 (0.0–0.0)	0.106∗
Duration of MAP values <65 mm Hg, min	1.0 (0.0–2.5)	1.4 (0.2–3.2)	0.052∗
Duration of MAP values <60 mm Hg, min	0.2 (0.0–0.3)	0.3 (0.0–1.8)	0.301∗
Duration of MAP values <50 mm Hg, min	0.0 (0.0–0.0)	0.0 (0.0–0.3)	0.080∗
Duration of MAP values <40 mm Hg, min	0.0 (0.0–0.0)	0.0 (0.0–0.0)	0.100∗
Absolute number of patients with any MAP value <65 mm Hg	90 (69)	107 (82)	0.022^†^
Absolute number of patients with any MAP value <60 mm Hg	74 (57)	81 (62)	0.451^†^
Absolute number of patients with any MAP value <50 mm Hg	33 (25)	47 (36)	0.081^†^
Absolute number of patients with any MAP value <40 mm Hg	11 (9)	20 (15)	0.125^†^
Absolute number of patients with MAP values <65 mm Hg for at least 1 continuous minute	55 (43)	57 (44)	0.901^†^
Absolute number of patients with MAP values <60 mm Hg for at least 1 continuous minute	34 (26)	37 (28)	0.781^†^
Absolute number of patients with MAP values <50 mm Hg for at least 1 continuous minute	11 (9)	10 (8)	0.824^†^
Absolute numbers of patient with MAP values <40 mm Hg for at least 1 continuous minute	2 (2)	4 (3)	0.684^†^

The median cumulative dose of norepinephrine patients were given was 0.9 (0.6–1.1) μg kg^−1^ in patients assigned to continuous norepinephrine infusion and 0.3 (0.2–0.4) μg kg^−1^ in patients assigned to manual bolus norepinephrine administration (*P*<0.001; estimated location shift 0.57 [95% CI 0.50 to 0.64] μg kg^−1^). In patients assigned to continuous norepinephrine infusion, the median norepinephrine infusion rate was 0.05 (0.03–0.07) μg kg^−1^ min^−1^, and the median number of norepinephrine infusion rate adjustments made was 2 (2–3). In patients assigned to manual bolus norepinephrine administration, the median number of norepinephrine boluses given was 2 (1–3), and the median norepinephrine bolus dose was 10 (10–10) μg.

The median area above a MAP of 100 mm Hg was 47.4 (8.7–131.1) mm Hg × min in patients assigned to continuous norepinephrine infusion and 42.0 (18.2–86.9) mm Hg × min in patients assigned to manual bolus norepinephrine administration (*P*=0.390; estimated location shift 4.11 [95% CI –5.79 to 18.79] mm Hg × min; Table 3).

**Table 3:** Secondary endpoints quantifying hypertension. Categorical data are presented as absolute number (percentage) and continuous data as median (25th–75th percentile). MAP, mean arterial pressure.∗*P*-values correspond to Wilcoxon rank-sum tests with continuity correction. ^†^*P*-values correspond to Fisher's exact test for count data.

Outcome	Continuous norepinephrine infusion (*n*=130)	Manual bolus norepinephrine administration (*n*=131)	*P*-values
Area above a MAP of 100 mm Hg, mm Hg × min	47.4 (8.7–131.1)	42.0 (18.2–86.9)	0.390∗
Area above a MAP of 110 mm Hg, mm Hg × min	14.1 (0.3–61.1)	13.4 (1.5–38.3)	0.703∗
Area above a MAP of 120 mm Hg, mm Hg × min	1.8 (0.0–20.2)	0.7 (0.0–12.7)	0.589∗
Area above a MAP of 140 mm Hg, mm Hg × min	0.0 (0.0–0.0)	0.0 (0.0–0.0)	0.489∗
Duration of MAP values >100 mm Hg, min	4.2 (1.6–7.7)	3.6 (2.4–5.3)	0.187∗
Duration of MAP values >110 mm Hg, min	2.0 (0.1–5.1)	1.8 (0.4–3.4)	0.499∗
Duration of MAP values >120 mm Hg, min	0.4 (0.0–2.8)	0.3 (0.0–1.6)	0.496∗
Duration of MAP values >140 mm Hg, min	0.0 (0.0–0.0)	0.0 (0.0–0.0)	0.465∗
Absolute number of patients with any MAP value >100 mm Hg	123 (95)	124 (95)	1.000^†^
Absolute number of patients with any MAP value >110 mm Hg	102 (79)	114 (87)	0.073^†^
Absolute number of patients with any MAP value >120 mm Hg	81 (62)	88 (67)	0.439^†^
Absolute number of patients with any MAP value >140 mm Hg	34 (26)	29 (22)	0.472^†^

## Discussion

We found that giving norepinephrine continuously via a syringe infusion pump during anaesthetic induction, compared with giving it as repeated manual boluses, did not reduce postinduction hypotension in low-to-moderate risk noncardiac surgery patients who had intermittent oscillometric blood pressure monitoring.

Our primary endpoint was the area under a MAP of 65 mm Hg, reflecting both the severity and duration of hypotension. The amount of postinduction hypotension was much lower than we had expected, even in patients assigned to manual bolus norepinephrine infusion. Perhaps, consequently, there was only a slight reduction in the median area under a MAP of 65 mm Hg of about 2 mm Hg × min (3.6 *vs* 5.5 mm Hg × min), an amount that was neither clinically meaningful nor statistically significant. For example, a difference of 2 mm Hg × min in the area under a MAP of 65 mm Hg might result from a MAP that was 59 mm Hg *vs* 61 mm Hg for 1 min during anaesthetic induction. Continuous, compared with manual bolus, norepinephrine administration also reduced the proportion of patients who had any MAP measurement <65 mm Hg from about 80% to 70% and the duration patients had MAP values <65 mm Hg from 1.4 to 1.0 min, reductions that again are not clinically meaningful.

Organ injury presumably accrues at hypotensive extremes.^16^ We thus also considered how many patients had any MAP measurement below various MAP thresholds. Low thresholds are especially interesting because available data suggest that most harm may accrue during brief periods of extreme hypotension.^16^ For instance, 11 (8%) of our patients assigned to continuous norepinephrine infusion and 20 (15%) patients assigned to manual bolus norepinephrine administration had any MAP measurement <40 mm Hg during induction of anaesthesia. Continuous norepinephrine infusion thus may have roughly halved the number of patients with most severe hypotension.

As might be expected, patients assigned to continuous norepinephrine infusion were given three times as much norepinephrine during induction of anaesthesia than patients assigned to manual bolus norepinephrine administration. A consequent concern is that continuous norepinephrine may have resulted in overtreatment and hypertension. However, median areas above a MAP of 100, 110, 120, and 140 mm Hg did not substantially differ between both groups. Furthermore, available evidence suggests that within broad limits intraoperative hypertension is minimally associated with organ injury,^17^^,^^18^ in contrast to hypotension which distinctly is.^19^

A strength of our trial was that we examined the increasingly common practice of administering norepinephrine through peripheral venous catheters. As in a cohort study of more than 14 000 patients,^20^ our experience is that continuous infusion of norepinephrine with a syringe infusion pump via peripheral venous catheters is effective and safe. A limitation of our trial is that we did not use a specific hemodynamic treatment protocol. Instead, clinicians were simply asked to keep MAP above 65 mm Hg to the best of their ability with the assigned norepinephrine administration approach. Outcomes with each approach thus critically depend on clinician responses, specifically how effectively they adjust the infusion rate or manage bolus administration.

Because of previous and ongoing studies and trials on blood pressure management,^15^^,^^21^, ^22^, ^23^ clinicians at our institution routinely administer norepinephrine during anaesthetic induction (either as manual bolus or by continuous infusion) and presumably treat hypotension rather aggressively. Norepinephrine management and the amount of hypotension thus likely differ in other centres. Norepinephrine is commonly used to treat postinduction and intraoperative hypotension in Europe.^24^ The choice of vasopressors may differ across regions and institutions, which limits the generalisability of our results. Moreover, our patient population was restricted to low-to-moderate risk patients. Anaesthesiologists could not be blinded to group allocation, which could have influenced the choice of drugs and doses. Statisticians were not blinded. Furthermore, the actual dropout rate slightly exceeded the anticipated rate, resulting in the final analysis including one patient fewer than originally planned in the sample size estimation. However, it is unlikely that this loss substantively altered our results.

In summary, giving norepinephrine continuously during anaesthetic induction, compared with giving it as repeated manual boluses, did not reduce postinduction hypotension in low-to-moderate risk noncardiac surgery patients who had intermittent oscillometric blood pressure monitoring. Clinicians can reasonably use either approach of giving norepinephrine to manage blood pressure during anaesthetic induction in low-to-moderate risk noncardiac surgery patients.

## Funding

This work was supported solely from institutional and/or departmental sources.

## Authors’ contributions

Study conception/design: KKT, BS

Study measurements: FK, KKT, DM, AK, MB

Data analysis/interpretation: all authors

Statistical analysis: KKT, LK, BS

Drafting of manuscript: KKT, DIS, BS

Critical revision of article for important intellectual content: all authors

Final approval of the version to be published: all authors

Agreement to be accountable for all aspects of the work thereby ensuring that questions related to the accuracy or integrity of any part of the work are appropriately investigated and resolved: all authors

KKT and BS had full access to all of the data in the study and are responsible for the integrity of the data and the accuracy of the data analysis.

## Declarations of interest

KKT has received honoraria for consulting and for giving lectures from Masimo (Neuchâtel, Switzerland). DIS is a consultant for Perceptive Medical (Newport Beach, CA, USA) and Dynocardia (Cambridge, MA, USA). BS is a consultant for Edwards Lifesciences (Irvine, CA, USA), Philips North America (Cambridge, MA, USA), GE Healthcare (Chicago, IL, USA), Maquet Critical Care (Solna, Sweden), Pulsion Medical Systems (Feldkirchen, Germany), Vygon (Aachen, Germany), Retia Medical (Valhalla, NY, USA), Masimo (Neuchâtel, Switzerland), Dynocardia (Cambridge, MA, USA). BS has received institutional restricted research grants from Edwards Lifesciences, Baxter (Deerfield, IL, USA), GE Healthcare, CNSystems Medizintechnik (Graz, Austria), Pulsion Medical Systems, Vygon, Retia Medical, Osypka Medical (Berlin, Germany). BS has received honoraria for giving lectures from Edwards Lifesciences, Philips Medizin Systeme Böblingen (Böblingen, Germany), Baxter, GE Healthcare, CNSystems Medizintechnik, Getinge (Gothenburg, Sweden), Pulsion Medical Systems, Vygon, Masimo, Ratiopharm (Ulm, Germany). BS is an Editor of the *British Journal of Anaesthesia*. FK, CV, LK, DM, MB, MW, AK, AB, and CZ have no conflicts of interest to declare.
