# Supplementary material for: Continuous versus bolus norepinephrine administration to treat hypotension after induction of general anaesthesia in low-to-moderate risk noncardiac surgery patients: a randomised trial
Source: Br J Anaesth. 2025 Feb 5;136(4):1137–44. doi: 10.1016/j.bja.2025.03.017 (PMC13014507; doi:10.1016/j.bja.2025.03.017)
Supplement: Multimedia component 1 [file mmc1.pdf]

Supplementary Figure S1: Exemplary illustration of the area under a mean arterial pressure of 65 mmHg. The red graph shows the mean arterial pressure over a period of 15 minutes. The area under a mean arterial pressure of 65 mmHg (red area below the dashed horizontal line) represents both the duration and severity of hypotension. MAP: mean arterial pressure.

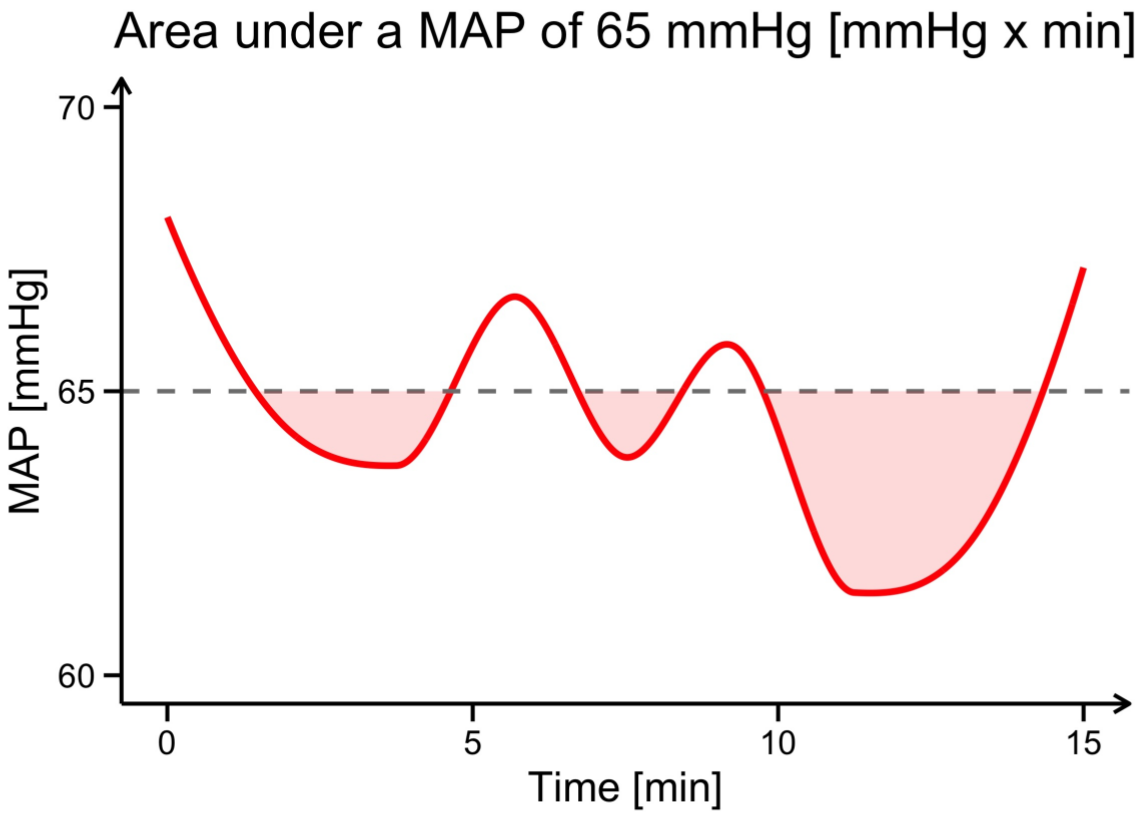

Supplementary Table S1 Anaesthetic management during induction

	**Continuous norepinephrine infusion (n=130)**	**Manual bolus norepinephrine administration (n=131)**
Preinduction neuraxial and peripheral nerve blocks, n	0 (0%)	0 (0%)
Endotracheal tube, n	66 (51%)	78 (60%)
Laryngeal mask, n	64 (49%)	53 (40%)
Propofol dose during induction, mg kg^{-1}	2.2 (1.9, 2.6)	2.1 (1.9, 2.5)
Sufentanil use, n	84 (65%)	74 (56%)
Sufentanil dose during induction, μg kg^{-1}	0.5 (0.4, 0.5)	0.4 (0.4, 0.5)
Remifentanil use, n	46 (35%)	57 (44%)
Remifentanil dose during induction, μg kg^{-1} min^{-1}	0.5 (0.5, 0.5)	0.5 (0.5, 0.5)

Data are presented as median (25th percentile, 75th percentile), or absolute number (percentage).
